# Anaphase Bridges: Not All Natural Fibers Are Healthy

**DOI:** 10.3390/genes11080902

**Published:** 2020-08-07

**Authors:** Alice Finardi, Lucia F. Massari, Rosella Visintin

**Affiliations:** 1 Department of Experimental Oncology, IEO, European Institute of Oncology IRCCS, 20139 Milan, Italy; alice.finardi@ieo.it; 2 The Wellcome Centre for Cell Biology, Institute of Cell Biology, School of Biological Sciences, University of Edinburgh, Edinburgh EH9 3BF, UK; lmassari@exseed.ed.ac.uk

**Keywords:** SCI, anaphase bridges, topoisomerases, catenane

## Abstract

At each round of cell division, the DNA must be correctly duplicated and distributed between the two daughter cells to maintain genome identity. In order to achieve proper chromosome replication and segregation, sister chromatids must be recognized as such and kept together until their separation. This process of cohesion is mainly achieved through proteinaceous linkages of cohesin complexes, which are loaded on the sister chromatids as they are generated during S phase. Cohesion between sister chromatids must be fully removed at anaphase to allow chromosome segregation. Other (non-proteinaceous) sources of cohesion between sister chromatids consist of DNA linkages or sister chromatid intertwines. DNA linkages are a natural consequence of DNA replication, but must be timely resolved before chromosome segregation to avoid the arising of DNA lesions and genome instability, a hallmark of cancer development. As complete resolution of sister chromatid intertwines only occurs during chromosome segregation, it is not clear whether DNA linkages that persist in mitosis are simply an unwanted leftover or whether they have a functional role. In this review, we provide an overview of DNA linkages between sister chromatids, from their origin to their resolution, and we discuss the consequences of a failure in their detection and processing and speculate on their potential role.

## 1. Sister Chromatid Intertwines: Structure, Origin and Resolution

**Figure 1:**
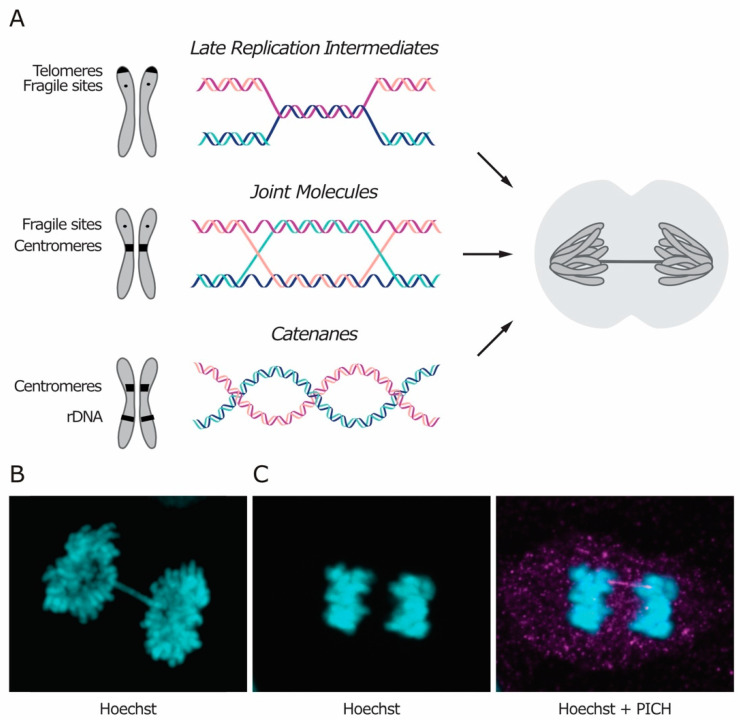
Three types of sister chromatid intertwines (SCIs) are observed during mitosis. (**A**) Molecular structures of late replication intermediates, joint molecules (here exemplified by a double Holliday junction) and catenanes are shown. Genomic loci at which each type of SCI were observed are indicated in the schematic chromosomes. The consequence of persistent SCIs in mitosis – a.k.a. anaphase bridges – is shown in the drawn cell. (**B**,**C**) Representative images of (**B**) a chromatin bridge and (**C**) an ultra-fine bridge are shown in retinal pigment epithelium (RPE-1) cells. DNA-intercalating dye Hoechst in cyan, Plk1-interacting checkpoint helicase (PICH) in magenta. Courtesy images by Neal Umbreit (**B**) and Mitchell Leibowitz (**C**).

Sister chromatid intertwines (SCIs) represent an additional source of cohesion between the chromatids. They arise during DNA replication and must be removed before cell division to preserve genome integrity. SCIs are usually classified based on their molecular structure, which reflects their origin. Three types of intertwines are recognized, namely short regions of un-replicated DNA, recombination intermediates and DNA catenanes (Figure 1A). Irrespectively of their nature and location in the genome, SCIs naturally arise during DNA replication and are mainly resolved in S phase, although some do persist and must be fully removed during mitosis to allow faithful chromosome segregation. Intertwines that persist through mitosis manifest themselves as DNA threads stretching between the two segregating DNA masses, known as anaphase bridges (Figure 1).

Although the number of anaphase bridges increases in the presence of stress, such as treatment with replication inhibitors or impairment of DNA repair pathways, DNA bridges are observed even in the absence of stressors and in untransformed cells, especially at the centromeres in mammalian cells [1,2]. In addition, anaphase bridges also form when cells attempt to segregate dicentric chromosomes that result from telomere-to-telomere fusion [3,4,5]. In contrast to the other classes of SCIs, which are thought to occur during normal DNA metabolism, telomere fusions are triggered by telomere disfunction, which can be caused by excessive telomere shortening or by malfunctioning of the sheltering complex that protects chromosome ends (reviewed in Reference [6]).

Anaphase bridges can be classified as either chromatin bridges or ultra-fine bridges (UFBs), based on whether or not the DNA is chromatinized (i.e., packaged with histones and other proteins) and can be stained with DNA dyes like Hoechst (Figure 1B,C). In unperturbed conditions, chromatin bridges are rare and, in yeast, they are mostly attributed to unresolved recombination intermediates [7]. On the other hand, UFBs can be observed in the majority of cells in anaphase, and they are enriched at the centromere in human cells [1,7]. The same kind of stressor can often induce formation of both types of bridges (i.e., chromatinized and non-chromatinized), suggesting that both can arise from the same kind of DNA intertwining. Currently, it is not clear what determines the chromatinized status of DNA bridges. Chromatin bridges may evolve into UFBs, according to the stage of their resolution. Alternatively, the chromatinized status of a bridge may be determined at the time of its formation. In the latter case, DNA chromatinization may depend on several factors, such as the extent of intertwining or the timing of recognition during mitosis.

Certain DNA intertwines are more commonly retained at specific loci. However, since different types of linkages can form anaphase bridges at the same region, their position in the genome is not sufficient to infer their molecular structure.

### 1.1. Incomplete Replication and Recombination Intermediates

Un-replicated regions provide a source of DNA linkages called late-replication intermediates (LRIs). LRIs are bonds between the two—otherwise fully replicated—sister chromatids and consist of short un-replicated double-stranded DNA (dsDNA) segments, where the two parental strands still anneal to each other. Since replication is generally completed well before anaphase, LRIs usually do not evolve into anaphase bridges. Instead, anaphase bridges can form in conditions of replication stress, preferentially at late replicating or hard-to-replicate regions, like common fragile sites (CFSs) [2]. Indeed, in human cells, replication of fragile sites is often left incomplete, particularly under conditions of replicative stress, leading to DNA damage in the next cell cycle [8,9]. Another region that poses a challenge to replication is the telomere, as it contains repeated DNA sequences and tends to form G-quadruplexes, secondary DNA structures that can stall the replication fork. Consistently, telomeric anaphase bridges increase if replication of these regions is impaired, for example by depletion of the Werner helicase, which contributes to the resolution of G-quadruplexes [10].

In yeast, replication of the highly repetitive and hard-to-replicate ribosomal DNA (rDNA) locus can continue up to metaphase [11]. Recently, it was also observed that replication of other specific regions (including sub-telomeric regions, fragile sites and other hard-to-replicate regions) is not completed until late anaphase, as demonstrated by the fact that inhibition of replication after metaphase hampered the resolution of chromatin bridges [12]. This observation indicates that, at least in yeast, some LRIs are taken care of through replication in late anaphase. This phenomenon may be a peculiarity of yeast cells, as in higher organisms LRIs have not yet been reported to turn into anaphase bridges in unperturbed conditions. A possible explanation for this discrepancy could be that, in yeast, G2 and metaphase are not clearly separated, while in humans, the better separation and the longer time provided between S phase and the onset of anaphase may be sufficient to allow completion of replication before chromosome segregation. However, in the presence of replicative stress, replication of CFSs is completed during early mitosis, also in human cells [13]. In this case, mitotic DNA synthesis fills the gaps and prevents un-replicated regions from bridging in anaphase [14,15].

Recombination intermediates are products of homologous recombination (HR), a DNA repair pathway which exploits the genetic information available on one sister chromatid to correct lesions in the other sister (reviewed in Reference [16]). In the presence of DNA damage, HR can be used to repair many types of DNA lesions, including double-strand breaks (DSBs). DNA repair through recombination mainly takes place in G2 and S phases and normally exploits the nearby sister chromatid to repair the lesion, although recombination between homologous chromosomes occasionally occurs in diploid cells. In addition to DNA repair, recombination is essential during replication for a process called template switching. In the presence of DNA lesions or stable DNA-protein complexes that halt fork progression, template switching allows the fork to restart and replicate across the lesion [17,18,19]. Recombination leads to the formation of a X-shaped structure called the Holliday junction, which covalently links the sister chromatids. The junction can be either single or double depending on whether only one or both ends of the DSB are invading the sister chromatid, respectively. The recombination process ends with the resolution of the generated joint molecules (JMs) through dedicated mechanisms. The simultaneous impairment of multiple pathways devoted to the resolution of JMs compromises chromosome segregation and cell viability in the absence of DNA damage [20,21,22,23]. This synthetic lethality indicates that HR-derived intertwines occur during every cell cycle, likely deriving from the use of template switching to rescue stalling of the replication fork.

It is not clear whether there are genomic regions where this type of DNA linkages preferentially arises. A study found UFBs from unresolved recombination intermediates in proximity to fragile sites and centromeres in human cells, after inducing anaphase bridges through depletion of the non-homologous end joining factor 53BP1 (p53 Binding Protein 1) [24]. The enrichment at CFSs and centromeres might reflect the fact that these regions represent a challenge to replication, maybe raising the need for HR to repair a possible lesion or to restore fork progression through template switching. However, another study performed on human cells impaired in the resolution of recombination intermediates found no colocalization between the resulting UFBs and centromere [20].

**Figure 2:**
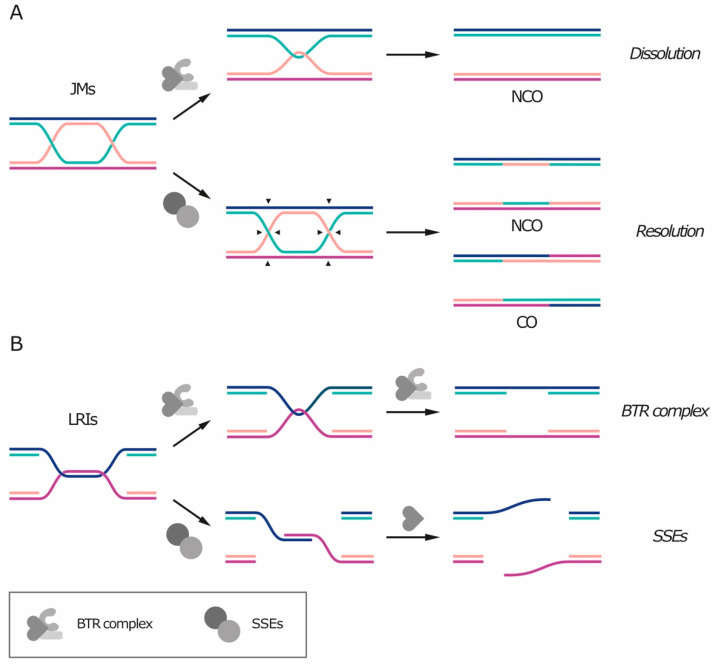
Joint molecules (JMs) and late replication intermediates (LRIs) can be disentangled by the BLM-TOP3A-RMI1/2 (BTR) complex or cleaved by structure-specific endonucleases (SSEs). (**A**) JMs can undergo BTR-mediated dissolution (top) or SSE-mediated resolution (bottom). Arrows indicate the possible sites of cleavage by nucleases. Dissolution always results in non-crossover products (NCO), whereas resolution can result in either crossover (CO) or non-crossover products. (**B**) LRIs can be processed by the BTR complex (top) or by structure-specific endonucleases (SSEs, bottom), leading to single-stranded DNA (ssDNA) gaps or ssDNA overhangs, respectively.

Un-replicated patches of DNA and double Holliday junctions share a common feature: they both contain—or can be converted into—a hemicatenane-like structure (Figure 2). Because of this similarity, these DNA linkages can be removed by the BTR/STR complex [20,25] (reviewed in Reference [26]). The BTR complex includes the RecQ helicase Bloom’s syndrome helicase (BLM), the topoisomerase TOP3A, and the single-stranded DNA (ssDNA) binding protein RMI1 or RMI2. The STR complex is its budding yeast counterpart and is composed by the Sgs1 helicase (orthologous to BLM), Top3 (orthologous to TOP3A) and Rmi1 (orthologous to RMI1). The processing of recombination intermediates through the BTR complex is termed “dissolution” [26] (Figure 2A, top). First, the helicase unwinds the heteroduplex, exposing ssDNA filaments. Top3 can then catalyze the strand passage reaction on the exposed ssDNA and remove the entanglement, while RMI1/2 stimulates supercoil relaxation and topoisomerase activity. As no nuclease is required and replication protein A (RPA) binds ssDNA and protects it from breaking [25], dissolution by the BTR complex does not create any additional DNA nick or gap. The hypothesis of BTR-mediated dissolution of the bridges is supported by the fact that BTR components localize on the bridges and that RPA is loaded on UFBs during anaphase in a BLM-dependent fashion [20].

Processing of LRIs by the BTR complex leads to the exposure of ssDNA segments as these regions were never replicated to begin with (Figure 2B, top). Thus, as the parental strands attempt to be separated, un-replicated regions form bridges that contain single-stranded DNA. If the exposure occurs at S phase, these gaps can be filled by replication; otherwise, they are thought to be repaired in the next cell cycle. The finding that DNA synthesis can occur also in mitosis (and, at least in yeast, as late as anaphase) raises the possibility that BTR-dependent disentangling of LRIs in mitosis might be followed by replication of the single-stranded regions. Indeed, in human cells, mitotic DNA synthesis was shown to be triggered by nuclease processing of fragile sites [14,15].

In the case of dissolution of JMs, after heteroduplex disentanglement, each filament returns to its complementary strand and the sister chromatids become physically separated. Dissolution of double Holliday junctions always generates non-crossover products (Figure 2A, top), meaning that there is no exchange of genetic information between the homologous chromosomes and that the DNA molecule is restored as it was before recombination [26].

Alternatively to the BTR complex, un-replicated segments and recombination intermediates can also be processed by dedicated structure-specific nucleases (SSEs), including MUS81-EME1/2 (Mus81-Mms4 in yeast), SLX1-SLX4, XPF-ERCC1 and GEN1 (Yen1 in yeast) (Figure 2A, bottom and Figure 2B, bottom, reviewed in References [27,28]). Simultaneous depletion of multiple SSEs is often synthetically lethal, indicating that they represent an essential pathway for the removal of recombination intermediates that are refractory to dissolution by the BTR complex, such as single or nicked Holliday junctions [29,30,31,32,33]. The removal of recombination intermediates through nuclease activity is called “resolution” and preferentially takes place in mitosis, while the BTR complex is active also in S phase (reviewed in Reference [34]). Differently from dissolution, SSE-mediated resolution can give rise to both crossover and non-crossover products, depending on how DNA filaments are cleaved (Figure 2A, bottom). If recombination occurs between homologous chromosomes, crossover events can cause loss of heterozygosity, which in turn can promote tumor development, thereby explaining why LRIs and JMs are preferentially processed by the BTR complex. In addition, premature SSE activation during S phase can target intermediates of DNA replication and repair for nucleolytic processing, with toxic effects such as chromosome pulverization [35]. Nucleases can also act in the next G1 and digest DNA bridges that persist after cell division, as shown for the cytoplasmic exonuclease TREX1 in mammalian cells [3].

While all anaphase bridges can break during cell division, leading to DNA lesions and chromosomal rearrangements, timely resolution of LRIs and JMs is of primary importance, as ssDNA is more fragile and sensitive to damaging agents.

### 1.2. Catenanes and Supercoils: A Race for Survival Devoid of Relaxation

DNA catenanes consist of dsDNA entanglements between the two sister chromatids and, unlike the types of intertwines previously described, catenanes represent pure topological linkages. The name “catenane” was introduced in early studies on circular DNA, where this type of intertwining results in catenation of two circular molecules [36], but is now used to also indicate entanglements between linear DNA.

**Figure 3:**
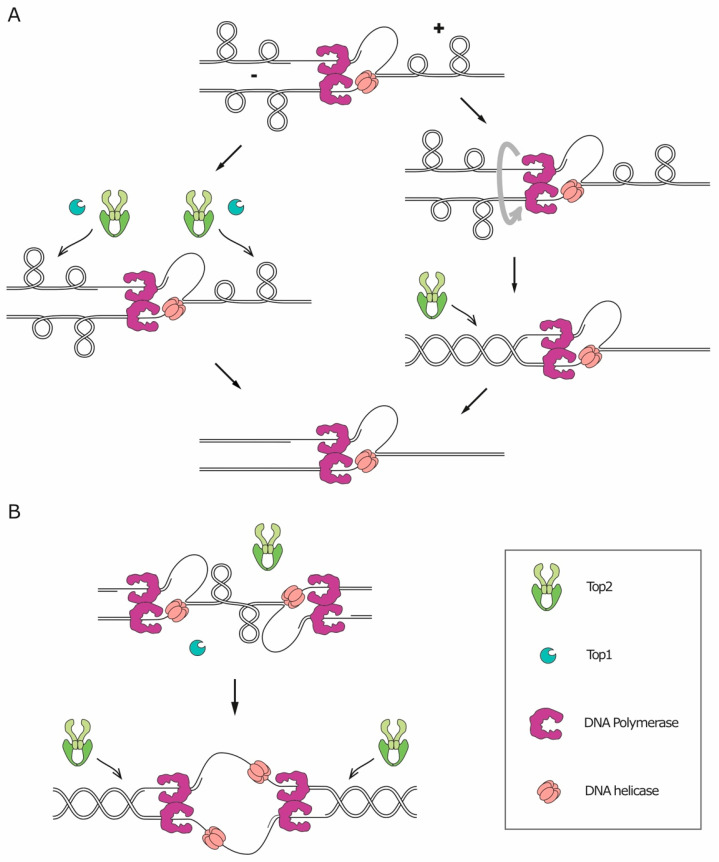
Relaxation of replication-induced supercoils occurs through topoisomerase action or fork rotation. (**A**) During replication elongation, positive and negative supercoiling accumulate ahead and behind of the replication fork, respectively. Replication-induced supercoil can be relaxed by both Top1 and Top2 (left). Alternatively, rotation of the replisome transfers the supercoiling accumulated ahead of the fork to the region behind the fork, determining the formation of precatenanes between the duplicated DNA molecules, which are resolved by Top2 (right). (**B**) At replication termination, the DNA between the two converging replisomes is inaccessible to topoisomerases. Therefore, fork rotation becomes the preferred route for the relaxation of replication-induced supercoils. Supercoils can be relaxed by either Top1 or Top2, whereas catenanes can only be removed by Top2.

The double-helical structure of DNA implies that any process that requires the opening of the two strands to access the genetic information (like replication, transcription, and recombination) will lead to topological issues, namely the accumulation of supercoiling. During replication/transcription, DNA helicases act ahead of the fork, catalyzing the opening of the double-helix and pushing the turns of the helix forward. The over-winding of the helix ahead of the fork is referred to as positive supercoiling, while the resulting underwinding behind the fork is called negative supercoiling (Figure 3). The topological tension imposed by the positive supercoiling needs to be relaxed, or it will eventually hinder helicase activity and stall the fork. In principle, on linear chromosomes, the supercoiling could diffuse along the molecule and be released by rotation around the axis. However, most eukaryotic chromosomes are extremely long DNA molecules, organized in domains, and contain many obstacles that can impede their axial rotation, such as DNA anchoring to the nuclear membrane, replication machineries and other DNA-protein complexes. In vivo, relaxation of supercoiling by axial rotation and diffusion along the chromosomes was shown to occur for shorter chromosomes [37,38] and to be favored by disruption of tethering to the nuclear membrane [39]. Axial rotation is, however, not sufficient to relax all the replication-induced supercoil. Therefore, to solve topological problems, cells need a set of dedicated enzymes, essential for DNA replication in all organisms, called topoisomerases (reviewed in Reference [40]). Topoisomerases can be divided into two different categories based on their molecular mechanism of action. Type I topoisomerases (Top1 and Top3) first catalyze the cleavage of one DNA strand, and then allow the other strand of the same DNA molecule to pass through the break and, finally, reseal the cleavage. Type II topoisomerases (Top2), instead, cleave both strands of a given DNA segment and then allow the passage of another double-stranded filament through the break, before ligating the cleaved segment. This characteristic of Top2 makes it the only enzyme capable of resolving catenanes [40].

During replication, the activity of topoisomerases is sufficient to relax the majority of the accumulated supercoils and to allow fork progression (Figure 3A, left). However, supercoils can also be relieved through an alternative mechanism which consists in the rotation of the replication fork around its axis, transferring the positive supercoiling from the un-replicated dsDNA ahead of the fork to the replicated dsDNA filaments behind the fork and resulting, therefore, in entanglements between the replicated sister chromatids (Figure 3A, right). Such structures are called precatenanes [41,42]. Fork rotation results in sister chromatid catenation, as precatenanes will eventually evolve into catenanes once replication is complete [43].

If fork rotation would relieve all supercoiling stress, one catenane would originate for each turn of the parental DNA helix. The relative rare occurrence of catenanes, compared with the number of turns of the helix [44], implies that fork rotation is not the preferred mechanism for supercoil relaxation. Several factors, indeed, limit the occurrence of fork rotation. First of all, it is reasonable to think that the bulky replisome itself constitutes a steric obstacle against its rotation. Secondly, fork rotation is actively counteracted by the replication pausing complex, Timeless/Tipin (Tof1/Csm3 in yeast) [45,46]. Thirdly, a recent in vitro study on the physical properties of chromatin demonstrated that a braided chromatin fiber is much stiffer than a single chromatin fiber, meaning that the duplicated DNA molecules behind the fork are more resistant to rotation than the un-replicated DNA molecule standing ahead of the fork. As a result, supercoiling is more easily translated ahead of the replication fork rather than behind it. In addition, Top2 was found to act more efficiently on single-fiber DNA supercoil than on precatenanes [47]. Altogether, these findings indicate that, during replication elongation, supercoiling is directed ahead of the fork, where topoisomerases can solve it with higher efficiency.

Fork rotation seems to be restricted to specific contexts, such as the sites of replication termination (Figure 3B). Early studies on replication in simian virus SV40 showed that the evolution of helical turns into catenanes occurs with low frequency during replication, with the exception of replication termination [44,48]. The formation of catenanes at the termination site is supported by the fact that Top2 is essential at replication termination, in both bacteria and eukaryotes [49,50,51,52,53]. A likely explanation is that the two converging replisomes prevent Top2 from accessing the DNA located between them, thus triggering fork rotation. The formation of catenanes at replication termination was also confirmed by a study in *Xenopus laevis*, showing that, at the termination site, the two converging replisomes pass by each other without stalling and lead to catenation of the replicated molecules [54]. In addition to termination sites, fork rotation seems to occur more frequently also at hard-to-replicate regions, as was shown in a study that evaluated the level of catenation retained on plasmids in yeast cells lacking Top2. Even though, in these cells, every fork rotation event translates into catenation of the plasmids, the number of catenanes was not greatly affected by the length of the replicated segment, indicating that fork rotation does not happen stochastically during elongation. Instead, the level of catenation increased if the plasmid contained regions known to pause replication, including transfer RNA genes (which are considered CFSs in yeast), inactive origins, and centromeres [45]. These observations suggest that fork rotation occurs preferentially at hard-to-replicate sites. This preference could be explained by the presence, at these regions, of physical barriers for the action of topoisomerases, such as stable DNA-protein complexes.

Although fork rotation may be more frequent at specific regions, an elegant study in yeast by Uhlmann and colleagues showed that the resulting catenanes are free to diffuse after replication, as their occurrence was the same throughout the chromosomes. This group developed a system based on excision and circularization of chromosomal segments, which allowed to obtain the first direct evidence of catenation in endogenous linear chromosomes [55]. The only region that was found to be devoid of catenanes is the silent mating-type locus, maybe because its high level of compaction increases the stiffness of chromatin, thereby preventing the intertwines from diffusing in this region. While the yeast mating-type locus is quite short, the same behavior is difficult to imagine for large heterochromatic regions in the mammalian genome, such as the ones that flank the centromeres.

## 2. SCI Resolution in Mitosis: Molecular Machineries and Their Mitotic Regulation

Albeit the majority of SCIs are resolved prior to chromosome segregation, cells have evolved specific strategies to remove the leftovers during mitosis. While LRIs and JMs rarely manifest themselves in normal mitosis, catenanes are found in most early anaphase cells and they account for the majority of DNA bridges in physiological conditions [1,56]. Complete resolution of catenanes only occurs concomitantly with chromosome segregation during a normal cell cycle and requires the activity of Top2 [50,57,58,59,60,61]. Several proteins have been suggested to play a role in the sensing and processing of DNA linkages in mitosis on the basis of their localization on anaphase bridges and of the accentuation of bridge formation following their impairment. However, it should be noted that many of these proteins are also involved in DNA condensation, replication, or repair, thus making it difficult to determine whether the increase in anaphase bridges that follows their impairment is due to a mitotic role or whether it is a consequence of defects earlier in the cell cycle.

### 2.1. The BTR Complex in Decatenation and Resolution

**Figure 4:**
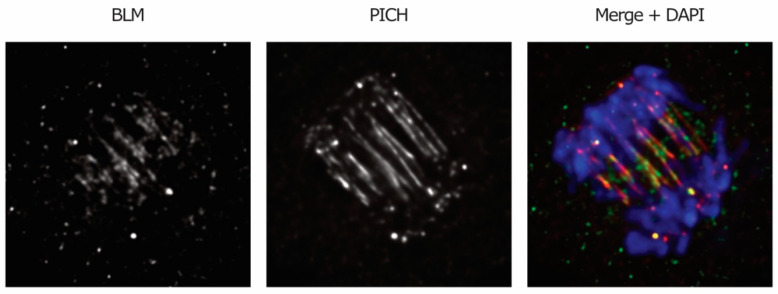
BLM and PICH associate with ultra-fine bridges (UFBs). UFBs were detected in RPE-1 cell treated with low doses of topoisomerase TOP2A inhibitor (ICRF193) by staining of BLM (in green) and PICH (in red). DNA was stained with 4′,6-diamidino-2-phenylindole (DAPI, in blue). Deconvolved images are shown. Courtesy images provided by María Fernández-Casañas.

A role for BLM helicase in the resolution of DNA linkages that persist in anaphase was suggested based on the findings that the components of the BTR complex are recruited to DNA bridges in anaphase (Figure 4) and that BLM-deficient cells display enhanced bridging [1]. A caveat for the latter observation is that the increase in anaphase bridges may also be due to the function of BLM earlier in the cell cycle, namely in DNA replication and repair (reviewed in Reference [26]).

The BTR complex can efficiently disentangle recombination intermediates and it was also proposed to act on other ssDNA-containing structures, for example un-replicated segments [10,25]. Furthermore, since it was shown to decatenate dsDNA in vitro, the BTR complex was also proposed to play a role in the resolution of catenanes [25,62], which is also supported by the observation that Top3 and Sgs1 can compensate for the lack of Top2 activity in the replication of the ribosomal DNA [63]. However, the BTR/STR complex cannot fully rescue the defects of Top2 mutants at later stages of mitosis, namely chromosome segregation errors and accumulation of DNA damage during mitosis. Therefore, while the BTR complex may possess decatenation activity and play a role in specific contexts, Top2 remains the main player resolving catenanes that persist in anaphase. Interestingly, BLM/Sgs1 was found to physically interact with Top2 in mitosis both in yeast and in human cells, and this interaction seems to be important for faithful chromosome segregation [64,65], although its significance is still unclear. BLM was found to be phosphorylated in a cell-cycle-dependent manner by cyclin-dependent kinase 1 (CDK1) and, likely, also by polo-like kinase 1 (PLK1), both in yeast and in human cells [66,67,68]. CDK-dependent phosphorylation stimulates BLM/Sgs1 activity in S-phase, while subsequent phosphorylation by Cdc5 (PLK1 homolog) may inactivate Sgs1 at metaphase [66], in line with the finding that the lack of PLK1 activity in mitosis leads to centromere disintegration and appearance of DNA threads reminiscent of UFBs in a BLM-dependent manner [69]. It is possible that, at metaphase, PLK1-dependent phosphorylation of BLM protects centromeres from unwanted helicase activity, to maintain the properties of centromeric chromatin and its ability to counteract the pulling force of the spindle.

### 2.2. Fanconi Anemia Proteins: Markers for Replication Intermediates and DNA Bridges

Fanconi Anemia (FA) proteins were originally identified through the search for mutations causing the genetic disease FA and include, among others, FANCD2, FANCI, and FANCM (reviewed in Reference [70]). Most of them are only found in mammals, although some (such as FANCD2 and FANCM) are conserved in all eukaryotes. FA proteins have been shown to play key roles in DNA repair and in response to replication stress. During S phase, they collaborate with other players (like the BTR complex) in the repair of DNA lesions that stall replication. The FANCD2-FANCI complex also protects stalled forks from degradation [71]. A role in the removal of DNA linkages in mitosis was suggested by the observations that some FA proteins localize on anaphase bridges and that FA-deficient cells display increased anaphase bridging [72,73,74]. FANCD2-FANCI foci mark UFBs that link fragile sites, which are thought to arise from incomplete replication, and, additionally, FANCD2-FANCI-associated bridges are enhanced by replication stress [2,73]. Conversely, the complex was not found on UFBs generated by inhibition of Top2 [2] or of the recombination pathway [20,24]. Altogether, these findings indicate that FANCD2-FANCI is a marker for LRI-derived bridges. As FANCD2-FANCI foci start appearing at fragile sites in S phase [2], it is not clear whether the complex actively participates in LRI resolution in mitosis or if it simply remains trapped at these regions. In conditions of replication stress, the frequency of BLM-associated UFBs is reduced in cells deficient in the FANC pathway compared with FANC-proficient cells, despite the overall amount of UFBs being similar [73]. This observation suggested that the FANC pathway may be involved in targeting BLM to LRIs in mitosis. Additionally, FANCM also associates to DNA bridges and its recruitment occurs during telophase in a BLM-dependent fashion [74]. Since FANCM possesses DNA translocase activity [75], this protein might directly participate in the removal of DNA bridges at late cell cycle stages.

### 2.3. Structure Specific Endonucleases at the Heart of Genome Integrity

SSEs act by cleaving DNA structures and represent an alternative strategy for the resolution of persistent late replication and recombination intermediates. The activity of these nucleases is mostly limited to mitosis and it is organized in two consecutive waves over time [34]. In particular, the activity of MUS81/Mus81-EME1/Mms4 and GEN1/Yen1 peaks at G2/M and anaphase, respectively. The distribution of activity in two peaks ensures the presence of one active nuclease throughout mitosis, and it may also be justified by differences in substrate specificity between the SSEs [27,28]. Notably, GEN1/Yen1, having broader substrate specificity, may represent the last chance to allow segregation, although with higher risk of genome rearrangements.

The regulation of SSE activity is coordinated with the cell cycle thanks to the action of mitotic kinases and phosphatases. In budding yeast, Cdc28 (the only Cdk) collaborates with Cdc5 to phosphorylate and activate Mus81-Mms4 in G2/M [76,77,78,79]. In S phase, DNA damage checkpoint activation leads to inactivation of Cdc5 and, as a consequence, also inhibits Mus81-Mms4 activity [78]. Concomitantly with Cdk and Cdc5 inactivation at anaphase onset, the activity of Mus81-Mms4 is gradually lost. Similarly, in human cells, CDK1 and PLK1 phosphorylate MUS81-EME1 and SLX4 in prometaphase, leading to the formation of the SMX tri-nuclease complex (composed by SLX1-SLX4, MUS81-EME1 and XPF-ERCC1), which can process Holliday junctions with much higher efficiency than single enzymes separately [31,35,80]. On the other hand, in yeast, Yen1 is kept inactive during S and G2/M phases through Cdc28-dependent phosphorylation [76], while, at anaphase onset, Cdc14 (the budding yeast Cdk-counteracting phosphatase) activates the nuclease by reversing this phosphorylation [81,82]. Yen1 phosphorylation not only reduces its catalytic activity, but also excludes it from the nucleus [81,83]. Regulation of GEN1/Yen1 through its localization is conserved in human cells where, because of its cytoplasmic localization, GEN1 only gains access to the DNA after nuclear envelope breakdown at the onset of mitosis [76,84].

The activity of nucleases was shown to promote mitotic DNA synthesis at hard-to-replicate regions. In human cells, in the presence of replicative stress, MUS81-EME1 and ERCC1 are recruited to fragile sites upon chromosome condensation, forming foci which disappear at the metaphase-to-anaphase transition [14,85,86]. Cleavage of fragile sites by MUS81-EME1 promotes repair of un-replicated regions through DNA synthesis in early mitosis [14]. Chromosome segregation in the absence of MUS81 or ERCC1 increments anaphase bridging at fragile sites, triggers DNA damage response in the next cell cycle, and causes chromosome segregation errors [14,85,86].

### 2.4. Resolving Catenanes: The Richer, the Better

Cells lacking Top2 activity display extensive DNA bridging in mitosis and lose viability in the next cell cycle [49,50,57,87,88]. Inhibition of Top2 triggers anaphase bridging at specific genomic loci, suggesting that DNA catenanes are preferentially retained at these regions. In higher eukaryotes, catenanes are by far most often retained on centromeric DNA [1,56,58]. One possible explanation for this feature is that, in higher eukaryotes, cohesin remains bound to these loci—but not to the chromosome arms—until the onset of anaphase [89] and interferes with decatenation by keeping sister chromatids close to each other. Consistently, the enrichment of catenanes at centromeres has not been detected in yeast, where cohesin also remains bound to the chromosome arms. An alternative explanation could be that centromeric heterochromatin reduces the accessibility of DNA to topoisomerases. In agreement with the latter hypothesis is the finding that Top2 is enriched not only at the centromeres but also at other heterochromatic regions in mammalian cells [90]. However, this explanation is at odds with the finding that the yeast mating-type locus is devoid of catenanes [55] and the fact that a certain level of chromosome compaction is required for decatenation. Moreover, the enrichment of Top2 on heterochromatin may also be due to the role of this enzyme in promoting DNA compaction [57,88,91,92].

During mitosis, Top2 associates dynamically with chromatin, and at metaphase, it is enriched at centromeres in mammals [88,90,93], consistently with the higher catenation of mammalian centromeres. In addition, another function of Top2 at centromeres was described. Top2 promotes the function of Aurora B [94] in the spindle assembly checkpoint (SAC)—a surveillance mechanism that ensures proper attachment of the chromosomes to the spindle (reviewed in Reference [95]). In particular, Top2 directly participates in Aurora B recruitment and activation at the centromeres through its interaction with Claspin and Haspin kinase, both of which are involved in the regulation of Aurora B [96,97,98].

In addition to centromeres, Top2 inhibition triggers anaphase bridging at telomeres [99] and at the nucleolus [100,101], indicating that these loci also retain catenanes in mitosis. Interestingly, Top2 is also required for proper segregation of the whole nucleolus, which happens late in anaphase both in yeast and in human cells [101,102]. In human cells, intertwining at rDNA loci was also observed between heterologous chromosomes in mitosis and disappeared at anaphase onset [103]. Intertwining between heterologous chromosomes is possible since, in higher eukaryotes, rDNA repeats are located on different chromosomes and positioned close to each other in the nucleolus. The accumulation of catenanes at the rDNA could be linked to the fact that this locus is highly transcribed during mitosis. Indeed, it is reasonable to think that transcription, by altering DNA topology, may interfere with topoisomerase activity at the fork, thus favoring replisome rotation and formation of catenanes. The impact of transcription on the formation of replication-induced SCIs is still largely obscure, however, and goes beyond the purpose of this review (for more information, we direct the interested reader to these reviews, References [104,105]).

The activity of Top2 in mitosis is regulated through post-translational modifications, including ubiquitination, phosphorylation, and conjugation with small ubiquitin-like modifier (SUMO, reviewed in Reference [88]), with the latter being the most extensively studied. SUMOylation of Top2 occurs at metaphase [106,107] and in yeast it is indirectly stimulated by Cdc5 [108]. Top2 was also found to be phosphorylated by PLK1 in human cells [109]. These findings, together, suggest a key role for this kinase in controlling Top2 in mitosis. SUMOylation directs Top2 recruitment to centromeres [110,111,112] and to the nucleolus [113]. In addition, SUMOylation of Top2 in mitosis guides its interaction with Claspin and Haspin, which in turn direct the recruitment of Aurora B to the centromeres [96,97,98]. While many studies have investigated the effects of SUMOylation on Top2 activity and association with chromatin, there is not a unified model for the effects of this modification on Top2 [96,107,112,114]. One possible reason for this discrepancy is the fact that different types of modifications on different residues may lead to distinct outcomes. In addition to post-translational modifications, an alternative mechanism for the recruitment of Top2 at metaphase, based on histone modifications, has recently been described in human cells. Phosphorylation of histone H2A, which is carried out by the SAC kinase Bub1 in proximity of the kinetochore, was shown to be necessary and sufficient for the recruitment of Top2 to the centromere, although evidence of direct interaction between the topoisomerase and H2A was not shown in the study [115].

### 2.5. TOPBP1/Dpb11: Damage Alert, Resolution, and Bridging

TOPBP1/Dpb11 is a protein involved in DNA damage checkpoint, replication, and repair, where it functions as a scaffold for protein recruitment [116,117,118,119,120,121]. During a normal mitosis, TOPBP1-foci form on chromatin upon mitotic entry and gradually disappear throughout mitosis [15]. In anaphase, TOPBP1 also associates with DNA bridges, especially UFBs [7,15,122]. TOPBP1 foci that persist throughout anaphase turn into 53BP1 foci in the next G1, indicating the presence of DNA damage. The gradual disappearance of TOPBP1 foci during mitosis probably reflects repair of SCIs, as supported by the fact that depletion of TOPBP1 after S phase enhances 53BP1 foci in the next cell cycle [15]. These observations suggested a role for TOPBP1 in the resolution of DNA linkages in mitosis. The fact that depletion of TOPBP1 leads to a reduction of UFBs and an accumulation of chromatin bridges [7], in addition to TOPBP1 localization on UFBs, may suggest a function in the dechromatinization of the bridges. Alternatively, these observations may be explained by TOPBP1 acting earlier in the cell cycle by suppressing HR, which normally gives rise to chromatin bridges [7].

TOPBP1 is also required for the recruitment of Top2 to anaphase bridges, indicating its involvement in the resolution of catenanes. Recruitment of TOPBP1 to the bridges and its interaction with Top2 both depend on the BRCA1 C Terminus (BRCT) domains of TOPBP1 [122]. As these domains usually recognize phosphorylated substrates, the recruitment of Top2 may depend on its phosphorylation. Finally, TOPBP1 also participates in the resolution of the other types of DNA intertwines in mitosis. In particular, TOPBP1 promotes both mitotic DNA synthesis at un-replicated fragile sites following replication stress [15] and resolution of recombination intermediates that arise from template switch, through its interaction with Slx1-Slx4 and Mus81-Mms4 [15,123].

### 2.6. PICH: The Sensor Sliding down Anaphase Bridges

Plk1-interacting checkpoint helicase (PICH) is a DNA translocase, meaning that it exploits adenosine triphosphate (ATP) hydrolysis to “walk” along DNA filaments [124], and it is only found in vertebrates and plants. Depletion of PICH was shown to cause displacement of the SAC protein Mad2 from kinetochores, thus compromising proper functioning of the checkpoint [56]. The idea that PICH also participates in the resolution of anaphase bridges originally came from the observation that it specifically localizes to DNA bridges in anaphase [56] (Figure 1C and Figure 4). In vitro experiments showed that PICH is able to bind dsDNA with an affinity that increases when the filaments are under stretching tension and then moves along the thread, sliding—and possibly removing—nucleosomes along the way [25,124,125]. The translocase activity, in addition to the fact that inhibition of PICH leads to an increase in chromatin bridges, but not UFBs [68,125,126], suggests that PICH might drive dechromatinization of the bridges. PICH was found to be associated with all kinds of UFBs at early anaphase and to be essential for the recruitment of other UFB-associated proteins, including the BTR complex [1,25]. Altogether, these findings point toward a role for PICH as an upstream factor and primary sensor of anaphase bridges. PICH collaborates with Top2 in the resolution of catenanes (before and after anaphase onset) and is able to stimulate the decatenating activity of the enzyme in vitro [126,127]. Recently, PICH was shown to induce an elevated degree of positive supercoiling by cooperating with the TOP3A-RMI1-RMI2 complex [128], which also localizes to UFBs [1]. This discovery provides a possible explanation for the role of PICH in the resolution of catenanes, as positive supercoiled DNA was shown to facilitate Top2-mediated decatenation [59].

## 3. Mitotic Events Involved in the Resolution of Catenanes

**Figure 5:**
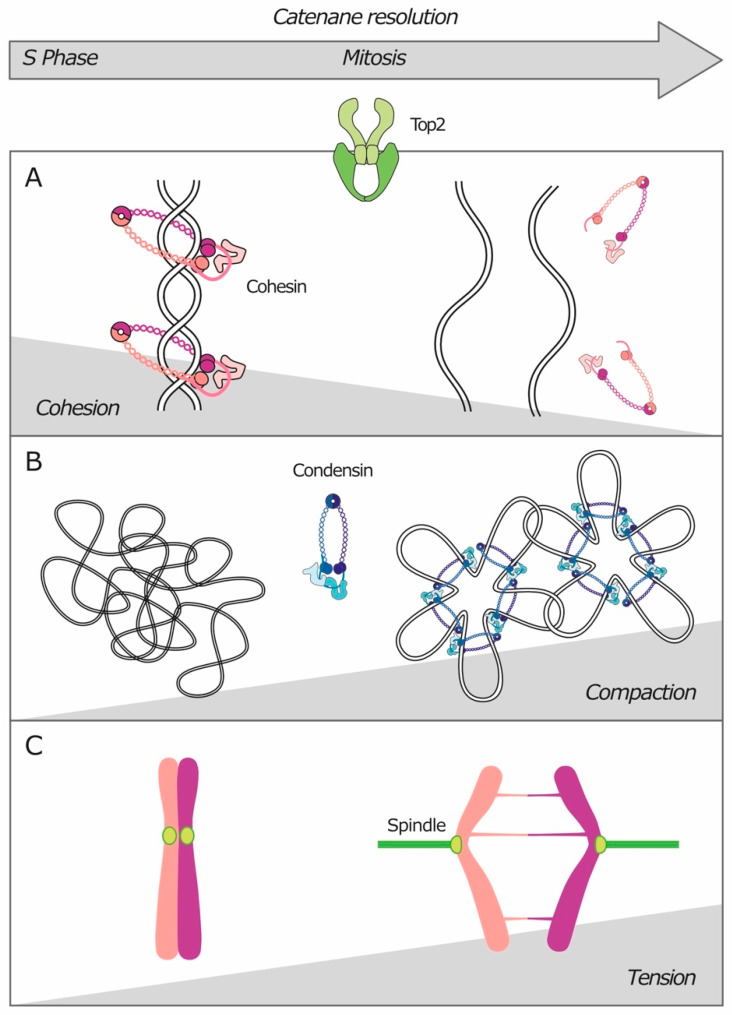
Chromatid individualization and separation aid the resolution of sister chromatid intertwines. During mitosis, (**A**) cleavage of cohesin complexes, (**B**) condensin-mediated compaction, and (**C**) chromatid separation by the mitotic spindle provide directionality to the reaction catalyzed by Top2, favoring the resolution of DNA catenanes.

As previously stated, most late replication and recombination intermediates are resolved before anaphase, while resolution of catenanes is normally completed concomitantly with chromosome segregation and it is influenced by other cellular processes (Figure 5).

Type II topoisomerases can both introduce and remove catenanes, depending on the direction of the thermodynamic equilibrium. In the pre-mitotic nucleus, where all duplicated chromatids are crowded together instead of being organized into mitotic chromosomes, Top2 may insert catenanes rather than removing them. This hypothesis is supported by the finding that Top2 can introduce de novo intertwining during a metaphase arrest if cells are treated with the microtubule-depolymerizing drug nocodazole [129] or if chromosome structure is disrupted [130]. To preserve genome stability, all SCIs must be properly resolved before exit from mitosis, which means going well below the thermodynamic equilibrium of the decatenation reaction. Even though, thanks to the energy provided by the ATP consumed during the reaction and the preference of the enzyme for bent DNA substrates, Top2 has the ability to decatenate DNA below the thermodynamic equilibrium, the mere action of the enzyme is not sufficient to explain the removal of every single catenane [131,132,133]. Here is where chromatid separation and individualization come to play a critical role. When chromatids undergo folding, they occupy a definite volume of space, separated from that of their sisters. In this way, all the SCIs are forced to remain at the interface between the sister chromatids, where the local concentration of catenanes increases, thus pushing the equilibrium of the Top2 reaction toward decatenation.

Chromatid individualization is a key step in the removal of all DNA linkages and other sources of cohesion, including cohesin complexes [134], and is achieved by compacting the chromosomes in a non-overlapping manner (reviewed in Reference [135]). The process is driven by structural maintenance of chromosome (SMC) complexes, namely cohesin, condensin, and Smc5/6. All SMC complexes have a ring-shaped structure, formed by two core SMC subunits and a kleisin subunit, and can link different DNA segments by embracing them within the ring (reviewed in Reference [136]). By binding DNA segments from the same chromosome, SMC complexes can drive chromosome condensation, through a mechanism called loop extrusion [136,137,138,139]. In addition, cohesin can embrace two DNA segments from sister chromatids, thus promoting sister chromatid cohesion. Cohesin, being the only complex that can induce both intra- and inter-molecular links, is responsible for chromosome cohesion, but is also implicated in modelling DNA architecture. On the other hand, condensin only promotes the formation of intramolecular links, thus being the main player in chromosome compaction and individualization. In budding yeast, cohesin and condensin both contribute to the shaping of mitotic chromosomes, although they play distinct roles [134,140,141], whereas in higher eukaryotes, the contribution of cohesin to mitotic chromosome condensation appears to be minimal. The function of the third SMC complex, the Smc5/6 complex, is much less understood. Smc5/6 can tether two DNA molecules [142], similar to cohesin, and it has essential functions in HR-mediated DNA repair, stabilization of stalled replication forks, and resolution of replication-induced topological stress (reviewed in Reference [143]). Smc5/6 was proposed to act at the replication fork and favor relaxation of supercoil by fork rotation, possibly by sequestering precatenanes forming behind the fork [38,142]. When Smc5/6 mutant cells undergo S phase, they accumulate unresolved recombination intermediates and replication-induced supercoil and fail to complete replication [144], leading to persistent SCIs that, in turn, compromise chromosome segregation and cause DNA damage. While the importance of Smc5/6 during S phase is well established, an essential role in the resolution of DNA linkages during mitosis has not been attributed to the complex yet [145,146].

Besides SMC complexes, chromatid individualization also requires Top2 activity [57,91,92,135], likely because the catalyzed strand-passage reaction is necessary to sustain the movement and reorganization of DNA fibers in space. Thereby, if, on the one hand, catenane resolution requires chromatid compaction, on the other hand, chromatid compaction requires Top2 activity. This interplay makes Top2 activity and SMC-dependent compaction two aspects of the same process: chromosome individualization.

In addition to individualization, the physical separation of chromatids aids the exposure and removal of any DNA linkages. Chromatid separation is driven by cohesin cleavage and by elongation of the mitotic spindle during anaphase.

### 3.1. Cohesin in a Heartfelt Embrace

Cohesin is a multi-protein ring-shaped complex, formed by the SMC proteins SMC1/Smc1 and SMC3/Smc3, and the kleisin subunits RAD21/Scc1 and SA/Scc3. As previously mentioned, the main role of this complex is to embrace the sister chromatids and to hold them together until their segregation (reviewed in Reference [147]). Although cohesin loading starts prior to DNA replication, in interphase, the complex tends to detach from DNA, while in S phase, the association between the complex and the DNA is stabilized through acetylation of Smc3, thereby establishing sister chromatid cohesion [148,149,150]. Cohesin cleavage is the key step that initiates chromosome segregation [151,152,153]. In yeast, the near totality of cohesin complexes is cleaved at anaphase entry, while in vertebrates, cohesin is removed from chromosome arms before metaphase and it is maintained up to anaphase onset only at the centromeres (reviewed in Reference [147]).

It is now well established that cohesin complexes favor Top2-dependent DNA catenation and prevent decatenation (Figure 5A). Early in vitro experiments showed that cohesin promoted catenation of DNA plasmids in the presence of Top2 [154]. The fact that the presence of cohesin bound to DNA prevents decatenation in vivo was first shown in yeast on DNA plasmids [60,61] and later confirmed also for endogenous chromosomes [55]. In higher eukaryotes, catenanes are specifically retained at centromeres, and they are resolved by Top2 only after that centromeric cohesin is cleaved prior to anaphase initiation [155]. The idea that cohesin is an obstacle for the removal of DNA linkages is also consistent with the fact that depletion of Wapl, a factor that destabilizes cohesin binding, promotes the formation of anaphase bridges [156,157]. Moreover, a study in *Drosophila* showed that heterochromatic segments ectopically placed on chromosome arms, which display cohesin enrichment starting from S phase, stretch abnormally during anaphase, despite the fact that cohesin has already been cleaved [158]. This finding suggests that the presence of cohesin before anaphase onset can hinder SCI resolution, to the point that sister chromatids remain linked even after cohesin cleavage. Rather than exerting an active role on Top2 or preventing its access to DNA, the negative effects of cohesin on decatenation seem to be due to the physical constraints imposed on sister chromatids. Cohesin complexes hold the sister chromatids in close proximity, thereby directing Top2 activity toward the insertion of catenanes rather than toward their removal. This model is supported by the finding that tethering DNA plasmids with means other than cohesin also increases the level of catenation [129].

Besides being responsible for sister chromatid cohesion through the formation of inter-molecular links, cohesin can also create loops within the same DNA molecule. In higher eukaryotes, this activity is mainly used in interphase to organize chromatin in topologically associating domains (reviewed in Reference [159]), while in yeast, it plays a major role in the condensation of chromosome arms in mitosis [140,141,160]. If cohesin is involved in condensation, it could also indirectly promote decatenation. This hypothesis is, however, difficult to test due to the current lack of experimental settings to distinguish between the cohesin complexes that hold together sister chromatids (promoting cohesion) and those that extrude DNA loops (promoting condensation).

### 3.2. Condensin: A Complex Compact Living

In vertebrates, there are two condensin complexes, condensin I and II, while budding yeast has only one complex, which is more similar to condensin I. Both complexes contain the two SMC subunits Smc2 and Smc4, but they differ in the kleisin subunit (CAP-H/Brn1 for condensin I, CAP-H2 for condensin II) and in the two HEAT repeats proteins (CAP-D2/Ycs4 and CAP-G/Ycg1 for condensin I, CAP-D3 and CAP-G2 for condensin II). The main function of condensin is to organize chromatin in a three-dimensional structure throughout the whole cell cycle (reviewed in References [135,161]). Initially, condensin was proposed to mediate DNA compaction by inducing positive supercoiling which, by wrapping the chromosome around itself, could promote its condensation and individualization [162,163]. However, it was later found that supercoiling is not the main fashion in which condensin compacts DNA [164,165]. Currently, the preferred model for mitotic chromosome condensation is based on condensin acting through loop extrusion [137,166,167].

Much evidence has been obtained for an interplay between condensin and Top2 in the resolution of sister chromatids. The first indication of the role of condensin in the resolution of DNA intertwines came from the finding that condensin mutants fail to properly segregate the chromosomes in mitosis and display anaphase bridges, similarly to Top2-deficient cells [168,169]. The interplay between condensin and Top2 has been extensively studied at the rDNA locus: Top2 and condensin are both required for proper segregation of the nucleolus and, indeed, mutations in either Top2 or condensin cause anaphase bridges at this locus [100,101,103,170]. These bridges can be resolved by ectopic expression of a viral topoisomerase II, demonstrating that they consist of DNA catenanes [102]. Further evidence on condensins role in SCI resolution came from studies on the catenation of yeast plasmids [59,61,129]. In particular, it was shown that, in the presence of a bipolar spindle, condensin induces extensive positive DNA supercoiling upon anaphase onset, thus determining Top2-dependent resolution of catenanes [59,129]. 

As for cohesin, the role of condensin in decatenation is mainly topological: by promoting chromosome condensation and individualization, condensin biases Top2 activity toward decatenation (Figure 5B). Final proof for this model is that removal of condensin from previously separated sister chromatids triggers their decompaction and Top2-dependent re-intertwining [130]. Whether condensin also has an additional, more direct effect on Top2 is still debated. In bacteria, the MukBEF complex, homologue to condensin, was found to physically interact with topoisomerase II [171]. If this interaction was conserved in eukaryotes, it could suggest a condensin-dependent recruitment of Top2 to regions that need to be decatenated, and it would also explain why, in yeast, Top2 was found to “follow” condensin when it relocates from centromeres toward chromosome arms after anaphase onset [172].

In yeast, where condensin and cohesin both contribute to mitotic chromosome condensation, condensin does not seem to affect the level of SCIs in G2/metaphase [55]. Instead, condensin appears to be required for completing chromatid separation specifically after anaphase onset [61,134], consistently with the finding that Cdc5-dependent phosphorylation stimulates condensin supercoiling activity in vitro [173]. As they gradually separate, sister chromatids stretch and recoil. The stretching behavior depends on the remaining cohesion between the arms of the two sister chromatids, mainly due to leftover cohesin complexes. The recoiling, instead, depends on condensin and is required to complete cohesin removal and sister chromatid separation [134]. Through the same mechanism, condensin-mediated recoiling could also drive the removal of other remaining sources of cohesion, namely, DNA linkages. Consistently, in mammalian cells, chromosome condensation was found to be maximized in anaphase [174]. 

### 3.3. Bipolar Spindles at Work

As spindle elongation and completion of SCI resolution occur in a concerted manner during anaphase, it is tempting to speculate that the former drives the latter. Most evidence for a role of the mitotic spindle in catenanes’ resolution comes from studies in yeast. It was first observed on DNA plasmids, and later confirmed on endogenous chromosomes, that treatment with nocodazole prevents decatenation at metaphase [55,59,60,61,129]. In particular, it was shown that catenanes are removed from centromeres only after their attachment to the metaphase spindle [55]. These findings demonstrate that chromosome attachment to a bipolar spindle is required for efficient resolution of SCIs, at least around the centromere in yeast. Moreover, spindle elongation and microtubule dynamics may aid the removal of catenanes also during anaphase. Indeed, in yeast, inactivation of the microtubule polymerase Stu2 impaired chromosome segregation, particularly in cells with abnormally long chromosomes. Segregation of these longer chromosomes is heavily dependent on Top2 activity in anaphase [39]. As chromosome segregation is partially rescued by removing chromosomal attachment to the nuclear membrane, these defects are attributed to a failure to resolve SCI during anaphase.

An intuitive explanation for these observations is that chromosome attachment to the spindle pulls apart the sister centromeres, allowing their physical separation, which in turn drives decatenation or simply helps intertwines to diffuse down along the chromosome (Figure 5C). By extension, it is likely that further spindle elongation after anaphase onset would assist the resolution of every single SCI also along the chromosome arms. In support of this hypothesis, in the presence of a functional condensin, spindle elongation in anaphase aids the removal of residual cohesin complexes [134]. In addition to chromosome separation, the tension imposed by the spindle could alter SCI topology and favor the action of the enzymes devoted to their recognition and resolution. This idea is consistent with the preference of PICH for stretched DNA substrates [25,124]. Also, the pulling force imposed by the spindle may unwind the hemicatenane-like portions of late replication and recombination intermediates, thus facilitating their dissolution by the BTR complex. Finally, the spindle could act indirectly through condensin. Indeed, bipolar spindle formation triggers the previously mentioned re-localization of condensin that occurs at anaphase, which in turn promotes Top2 recruitment to the chromosome arms [172].

Alternatively, the elongating spindle might act as a ruler and directly regulate the completion of SCI resolution. Interestingly, the idea of the spindle acting as a ruler, coordinating cellular events in space and time, has already been shown. The mechanism relies on the association of Aurora B with the spindle midzone in anaphase, which creates a phosphorylation gradient that fades toward the poles of the cell [175]. This Aurora B gradient controls events like anaphase chromosome compaction, spindle disassembly, and mitotic exit, coordinating them with spindle elongation [174,176,177,178]. The aim of this mechanism seems to be to physically separate the chromosomes and to clear the way for cytokinesis, to avoid trapping and breaking of DNA. Consistently, yeast cells divide with longer spindles if condensation is compromised or if the chromosome arm length is increased [102,158].

## 4. Anaphase Bridges at the Heart of Genome (In)Stability

### 4.1. A Time to Bridge and a Time to Rupture

Persistent anaphase bridges, independently of the source, can cause gross chromosomal rearrangements, a hallmark of cancer development. Chromosomal rearrangements are likely a consequence of DSBs that are formed when the bridge eventually breaks. Indeed, it was recently shown that even one single unresolved bridge triggers a cascade of events that result in increasing amounts of genome rearrangements [5]. Highly-proliferating cancer cells are known to be under increased replicative stress [179] and, since replicative stress enhances anaphase bridges [2,7,73], it is reasonable to think that these structures may arise in cancer cells with a higher frequency compared to normal conditions. If this scenario was true, anaphase bridges, being both a consequence of replicative stress and a cause of genome instability, could be considered a driver of tumor progression.

Several models have been proposed to explain how anaphase bridges drive genomic instability. The first model is the so-called breakage-fusion-bridge (BFB) cycle, described by Barbara McClintock [180]. According to this idea, DNA bridges that are cleaved during cytokinesis are re-sealed in the daughter cells with other chromosome pieces, causing gross genome rearrangements in the form of reciprocal exchanges of chromosome arms. The BFB model was recently extended by Pellman and colleagues. In particular, they found that bridge rupture, in addition to causing the chromosomal aberrations predicted by the BFB model, also leads to more complex rearrangements, namely chromothripsis [5]. Chromothripsis is the shattering of one or a few chromosomes, followed by stitching of the fragments in a random order. Chromothripsis is commonly observed starting from the second generation after bridge occurrence, and it is caused by abnormal replication of the bridging DNA during mitosis [5]. In addition to BFB, Chan and colleagues proposed an alternative model for the rupture of UFBs arising from recombination intermediates, called sister-chromatid rupture and bridging [24]. In this case, in contrast to BFB, the cleavage occurs shortly after anaphase onset and is independent of cytokinesis. According to this model, the intertwined sister chromatids break along the chromosome axis at the site where the bridge originates, while the bridge itself remains intact. As a result, the two sister arms are fused through the bridge and are thereby inherited by one of the two daughter cells. This mechanism is predicted to lead to specific chromosome rearrangements, including whole-arm deletions and translocations.

How do anaphase bridges break? It seems reasonable to think that threads of uncompacted—or even unchromatinized and single-stranded—DNA are fragile and can easily be broken by spindle elongation or by the actomyosin ring during cytokinesis. Although the pulling force generated by the spindle is estimated to be far too weak to break mitotic chromosomes [181], it could be sufficient to break a single DNA thread [182]. Therefore, spindle elongation may be able to break anaphase bridges, especially if they contain ssDNA segments, such as the ones generated by the processing of JMs and LRIs. Nevertheless, strong evidence of DNA linkages broken in this fashion in vivo is currently lacking. Instead, there is evidence for cytokinesis being able to sever anaphase bridges. For example, cytokinesis was proven to be responsible for the breakage of chromatin bridges in Top2 and condensin yeast mutants [52,183].

In order to prevent the severe DNA damage caused by rupture of DNA threads during cell abscission, cells have evolved a mechanism that delays cytokinesis in the presence of chromatin trapped on the site of cleavage. In yeast cells, this mechanism is termed the NoCut checkpoint, and it requires the activity of Aurora B/Ipl1 at the spindle midzone [184,185]. The NoCut is sensitive to SCIs coming from various sources, including replicative stress and lack of condensin or Top2 activity, but it fails to detect bridging from dicentric chromosomes [186]. Aurora B acts as a sensor for chromatin at the cleavage plane and, in the presence of DNA bridges, inhibits completion of abscission and stabilizes the spindle [184,185,186]. The activation of the NoCut checkpoint could provide additional time for the removal of the bridges in anaphase, although cells eventually divide if the bridge cannot be resolved.

In yeast, cytokinesis always leads to rupture of the anaphase bridges, likely because the yeast cell wall represents an impenetrable barrier for DNA filaments. Conversely, in mammalian cells, cytokinesis in the presence of unresolved bridges can have different outcomes, all of which can jeopardize genomic stability. First, as already mentioned, bridges can be cleaved during abscission, causing DSBs [3]. The second option is furrow regression [187]. In this case, although ingression of the cleavage furrow is initiated, cell division is never completed and results in tetraploidy, a condition that can cause senescence/apoptosis or, in some cases, initiate genomic instability. Thirdly, a mechanism reminiscent of the yeast NoCut checkpoint, called the abscission checkpoint, can come into play (reviewed in Reference [188]). Similar to the NoCut, the abscission checkpoint relies on Aurora B at the cleavage site, acting as a sensor of chromatin and delaying abscission in the presence of DNA bridges. Activation of this checkpoint triggers abscission failure and bridge stabilization, thus preventing furrow regression and tetraploidization. In the case of abscission failure, after cytokinesis, the daughter cells remain linked by the intact DNA thread, but behave like separate identities [187]. Indeed, in human cells, chromatin bridges rarely break during anaphase spindle elongation and they are often retained until the next G1 [3,187,189]. However, even in this case, the bridge eventually breaks and, while the nuclease TREX1 was suggested to participate in this process [3], the main factor determining the rupture of the bridge seems to be the mechanical stretching imposed by the daughter cells migrating away from each other [5]. Irrespective of the mechanism, once again, the rupture of the bridge promotes chromosomal rearrangements and chromothripsis [3,5].

### 4.2. Footprints Leading Back to the Old Days

Given how dangerous anaphase bridges can be for genomic integrity, one may wonder if a mitotic checkpoint exists that monitors the level of DNA intertwining. Catenanes do not trigger the DNA damage checkpoint and they also fail to activate the SAC, meaning that they do not alter the tension properties of the chromosome attachment to the spindle. The existence of a decatenation checkpoint, which delays entry into mitosis in response to extensive DNA catenation, was first suggested by the observation that drugs that inhibit Top2 can delay the G2/M transition in mammalian cells [190,191]. However, it was later argued that Top2 poisons can trigger the DNA damage checkpoint and the p38-stress-activated checkpoint [192,193]. Therefore, the existence of a checkpoint protecting against extensive SCI remains a controversial topic. In the future, to solve the controversy, it could be useful to adopt genetic approaches, to avoid the possible off-target effects of Top2 poisons.

Independently of the existence of a decatenation checkpoint, persistent DNA linkages appear to be features of a normal cell cycle, raising the question as to whether they simply cannot be resolved before chromosome segregation or whether they play a beneficial role. Catenation could serve as another mechanism of cohesion between sister chromatids. Indeed, centromeric intertwines are sufficient to prevent sister chromatid segregation in the absence of cohesin complexes [155]. Moreover, *Drosophila* cells lacking either condensin or Top2, both of which display extensive SCIs, are able to bypass the SAC arrest triggered by depletion of the cohesin subunit RAD21 and segregate their chromosomes [94]. These findings demonstrate that DNA linkages, like the cohesin complex, can provide cohesion, but do not imply that this feature is actually exploited by cells. The fact that cohesin is the main source of cohesion and that mitotic factors are necessary for catenane resolution argues that persistent SCIs are simply a leftover that cannot be removed earlier.

Clarke and colleagues suggested that, from an evolutionary perspective, DNA linkages may have represented the primary source of cohesion before cohesin appeared [194]. We find this hypothesis intriguing for several reasons. First, while cohesin must be actively loaded on the chromosomes, SCIs arise concomitantly with DNA replication as a natural consequence. Secondly, in bacteria, similar to eukaryotes, DNA molecules retain DNA linkages after duplication: they can be catenated or even interconnected upon recombination (reviewed in Reference [195]). Thirdly, bacteria do not seem to have any protein complexes devoted to maintaining cohesion between the sister DNA molecules. Indeed, no cohesin homologue has been found in prokaryotes, although a SMC homologue to eukaryotic condensin is present in many species. This absence is likely explained by the fact that there is no need for sister chromatid cohesion in bacteria, since chromosome segregation and DNA duplication occur simultaneously.

In eukaryotes, the genome is more complex, with multiple linear chromosomes. In addition, DNA replication and mitosis occur at distinct times, raising the need to ensure that sister chromatid cohesion is maintained until all chromosomes can be segregated in a concerted manner, to avoid cell division with unequal distribution of the genome. While cohesin likely evolved to satisfy this requirement, SCIs could have had a more prominent role before cohesin appeared. In modern organisms, DNA intertwines may still play an accessory role in particular conditions or at certain genomic regions. For example, the additional cohesion provided by catenanes may be exploited to control the time of segregation of different genomic loci. Interestingly, in yeast cells, decatenation and segregation of the nucleolus, which normally occur late in anaphase, is triggered by Cdc14-dependent condensin recruitment to this locus at anaphase onset [170].

Whether or not SCIs had a function at some point during evolution, the strategies for their resolution appear to be conserved. Indeed, both in bacteria and eukaryotes, chromosome compaction aids chromosome segregation. Moreover, in both domains, the resolution of DNA linkages occurs at the final steps of segregation and is aided by the forces that pull the sister chromosomes toward the opposite cell poles. The evolutionary conservation of the strategies for SCI resolution points to the fact that DNA linkages, being a natural consequence of DNA replication, likely existed (and have been threatening genome stability) since the early days of life.

## Abbreviations

CFS	Common fragile site
DSB	Double-strand break
dsDNA	Double-stranded DNA
FA	Fanconi Anemia
HR	Homologous recombination
JM	Joint molecule
LRI	Late-replication intermediate
rDNA	Ribosomal DNA
SAC	Spindle assembly checkpoint
SCI	Sister chromatid intertwine
ssDNA	single-stranded DNA
SSE	Structure-selective endonuclease
UFB	Ultra-fine DNA bridge
